# Interleukin-10 Attenuates Hypochlorous Acid-Mediated Cytotoxicity to HEI-OC1 Cochlear Cells

**DOI:** 10.3389/fncel.2017.00314

**Published:** 2017-10-06

**Authors:** Martin Mwangi, Sung-Hee Kil, David Phak, Hun Yi Park, David J. Lim, Raekil Park, Sung K. Moon

**Affiliations:** ^1^Department of Head and Neck Surgery, David Geffen School of Medicine, University of California, Los Angeles, Los Angeles, CA, United States; ^2^Department of Otolaryngology, Ajou University School of Medicine, Suwon, South Korea; ^3^Gwangju Institute of Science and Technology, Gwangju, South Korea

**Keywords:** oto-protection, IL-10, IL-22, hypochlorous acid, HMOX1, Nrf2, NR0B2

## Abstract

Inflammatory reaction plays a crucial role in the pathophysiology of acquired hearing loss such as ototoxicity and labyrinthitis. In our earlier work, we showed the pivotal role of otic fibrocytes in cochlear inflammation and the critical involvement of proinflammatory cytokines in cisplatin ototoxicity. We also demonstrated that otic fibrocytes inhibit monocyte chemoattractant protein 1 (CCL2) upregulation in response to interleukin-10 (IL-10) via heme oxygenase 1 (HMOX1) signaling, resulting in suppression of cochlear inflammation. However, it is still unclear how IL-10 affects inflammation-mediated cochlear injury. Here we aim to determine how hypochlorous acid, a model inflammation mediator affects cochlear cell viability and how IL-10 affects hypochlorous acid-mediated cochlear cell injury. NaOCl, a sodium salt of hypochlorous acid (HOCl) was found to induce cytotoxicity of HEI-OC1 cells in a dose-dependent manner. Combination of hydrogen peroxide and myeloperoxidase augmented cisplatin cytotoxicity, and this synergism was inhibited by N-Acetyl-L-cysteine and ML-171. The rat spiral ligament cell line (RSL) appeared to upregulate the antioxidant response element (ARE) activities upon exposure to IL-10. RSL cells upregulated the expression of NRF2 (an ARE ligand) and NR0B2 in response to CoPP (a HMOX1 inducer), but not to ZnPP (a HMOX1 inhibitor). Adenovirus-mediated overexpression of NR0B2 was found to suppress CCL2 upregulation. IL-10-positive cells appeared in the mouse stria vascularis 1 day after intraperitoneal injection of lipopolysaccharide (LPS). Five days after injection, IL-10-positive cells were observed in the spiral ligament, spiral limbus, spiral ganglia, and suprastrial area, but not in the stria vascularis. IL-10R1 appeared to be expressed in the mouse organ of Corti as well as HEI-OC1 cells. HEI-OC1 cells upregulated Bcl-xL expression in response to IL-10, and IL-10 was shown to attenuate NaOCl-induced cytotoxicity. In addition, HEI-OC1 cells upregulated IL-22RA upon exposure to cisplatin, and NaOCl cytotoxicity was inhibited by IL-22. Taken together, our findings suggest that hypochlorous acid is involved in cochlear injury and that IL-10 potentially reduces cochlear injury through not only inhibition of inflammation but also enhancement of cochlear cell viability. Further studies are needed to determine immunological characteristics of intracochlear IL-10-positive cells and elucidate molecular mechanisms involved in the otoprotective activity of IL-10.

## Introduction

Inflammation is a tightly controlled process because excessive inflammation potentially leads to unintended tissue injury. In the cochlea, inflammation is increasingly recognized to contribute to the pathophysiology of acquired sensorineural hearing loss (SNHL) such as ototoxicity, given that lipopolysaccharide (LPS)-induced inflammatory response aggravates cisplatin ototoxicity as well as the synergistic ototoxicity of kanamycin and furosemide (Oh et al., 2011; Hirose et al., 2014). Yet, it has not been fully understood how inflammatory reaction itself induces cochlear injury.

Pro-inflammatory cytokines such as TNF-α are known to critically mediate cisplatin ototoxicity (So et al., 2007, 2008), but the cochlear sensory cells appeared to be damaged only by the extremely high concentrations of TNF-α in animal experiments (Dinh et al., 2008; Keithley et al., 2008), indicating the involvement of multiple factors in inflammation-mediated cochlear injury. Among a number of inflammatory mediators, hypochlorous acid (HOCl) has gained attention due to the essential contribution to tissue injury (Johnson et al., 1987; Hammerschmidt and Wahn, 1997). Hypochlorous acid is a potent oxidant, released from activated phagocytes during the respiratory burst for the destruction of invading pathogens. Due to its powerful oxidative property, there is a risk of host tissue injuries when associated with excessive inflammatory reactions (Pullar et al., 2000). However, it is unclear whether hypochlorous acid is ototoxic and contributes to inflammation-mediated cochlear injury.

In our earlier work, downregulation of proinflammatory cytokines appeared to attenuate cisplatin ototoxicity (So et al., 2008), which led us to focus on the anti-inflammatory cytokine, IL-10. Otic fibrocytes were shown to inhibit monocyte chemoattractant protein-1 (CCL2) upregulation in response to IL-10 *via* heme oxygenase 1 (HMOX1) signaling, resulting in suppression of cochlear inflammation. However, it is unclear how IL-10 maintains HMOX1 upregulation because IL-10 paradoxically inhibits p38 MAPK that is required for HMOX1 upregulation (Kontoyiannis et al., 2001). Based on the finding showing the involvement of NRF2 (also known as NFE2L2) in HMOX1 regulation in cisplatin ototoxicity (So et al., 2006), we aim to elucidate an NRF2-mediated alternative pathway maintaining IL-10-induced HMOX1 regulation. Furthermore, NRF2 is involved in the regulation of NR0B2 (Huang et al., 2010), an orphan nuclear receptor involved in negative regulation of inflammatory reactions through inhibition of NF-κB (Yuk et al., 2011). Thus, we hypothesize that NR0B2 contributes to the anti-inflammatory effect of IL-10 on cochlear inflammation.

Besides the anti-inflammatory activity, there is accumulating evidence showing the cytoprotective activity of the IL-10 family cytokines. It has been reported that IL-10 upregulates anti-apoptotic factors such as Bcl-2 and Bcl-xL (Levy and Brouet, 1994; Stassi et al., 2000) and enhances cell viability of cortical neurons and retinal ganglion cells (Boyd et al., 2003; Sharma et al., 2011). Moreover, IL-22, which shares IL-10R2 with IL-10 for forming an active IL-22R complex, promotes the survival of hepatocytes (Radaeva et al., 2004) and even upregulates IL-10 in colon epithelial cells (Nagalakshmi et al., 2004). Moreover, IL-22 contributes to mucosal wound healing and intestinal epithelial regeneration via STAT3 signaling (Pickert et al., 2009; Lindemans et al., 2015). Based on these findings, we aim to determine cytoprotective activities of IL-10 and IL-22, inhibiting cochlear injury through promoting cochlear cell viability.

Here, we demonstrate that hypochlorous acid not only reduces cochlear cell viability but also exacerbates cisplatin ototoxicity, and that IL-10 is protective for hypochlorous acid-induced cytotoxicity. We found cochlear localization of IL-10-expressing cells and IL-10R1 expression in the organ of Corti. Moreover, it was shown that NRF2 and NR0B2 contribute to the IL-10 signaling network and that HEI-OC1 cells upregulate Bcl-xL expression in response to IL-10. This study may enable us to better understand the molecular pathogenesis involved in inflammation-mediated cochlear injury and would provide a scientific basis for the development of therapeutic tools to manage acquired SNHL.

## Methods

### Reagents

Sodium hypochlorite (NaOCl), cisplatin (*cis*-[Pt(NH_3_)_2_(Cl)_2_]), protoporphyrin IX cobalt chloride (C_34_H_32_CoN_4_O_4_Cl, CoPP), protoporphyrin IX zinc(II) (C_34_H_32_N_4_O_4_Zn, ZnPP), N-Acetyl-L-cysteine, LPS, recombinant IL-10, IL-22, and myeloperoxidase were purchased from Sigma-Aldrich (St. Louis, MO). ML-171 was purchased from Tocris (Minneapolis, MN). TaqMan primers and probes for rat CCL2 (Rn00580555_m1), rat HMOX1 (Rn01536933_m1), rat NRF2 (Rn00582415_m1), rat NR0B2 (Rn00589173_m1), and rat GAPDH (4352338E) were purchased from Life Technologies (Grand Island, NY).

### Animals and immunolabeling

Young adult C57BL/6 mice (The Jackson Laboratory, Bar Harbor, ME) were used. All animal experiments were approved by the Institutional Animal Care and Use Committee of University of California, Los Angeles. To induce cochlear inflammation, animals were injected i.p. with a non-septic dose (1 mg/kg) of LPS (Sigma-Aldrich) and euthanized 1 or 5 d after injection. Control animals were given with normal saline. For temporal bone sections, mouse temporal bones were dissected after decapitation. After fixation, decalcification and embedding in paraffin, serial sections (~10 μm thickness) were prepared through the mid-modiolar plane and were used for immunolabeling of IL-10 or IL-10R1. For whole mount preparation, bony otic capsules were carefully removed, and cochlear lateral wall tissues were dissected as described (Moon et al., 2007) and were further used for IL-10 immunolabeling. Cochlear lateral tissues were fixed in 4% paraformaldehyde overnight at 4°C and permeabilized in 0.5% Triton X-100 (Sigma-Aldrich) for 1 h. After immunoblocking with 10% goat serum, samples were incubated with a rat antibody against IL-10 (1:100, Santa Cruz Biotechnology, Dallas, TX) or IL-10R1 (1:200, Thermo Scientific, Waltham, MA) overnight at 4°C. After washing, sections were incubated with rhodamine-conjugated goat anti-rat IgG (1:200, Life Technologies). Mounted with anti-fade mounting media (Life Technologies), samples were viewed and photographed using a TCS SP5 confocal microscope (Leica, Buffalo Grove, IL).

### Cell culture, cell viability assays, and quantitative RT-PCR

Rat spiral ligament fibrocyte cell line (RSL) were maintained in DMEM supplemented with 10% FBS, penicillin (100 U/ml), and streptomycin (0.1 mg/ml; Life Technologies) at 37°C in a humidified atmosphere of 5% CO_2_ and 95% air as described (Yian et al., 2006). HEI-OC1 cells were cultured under a permissive condition (33°C, 10% CO_2_) in high-glucose DMEM containing 10% FBS without antibiotics as described (Kalinec et al., 2003). For cell viability, HEI-OC1 cells were exposed to cytotoxic agents such as NaOCl (1:50~1:1000), cisplatin (100 μM), hydrogen peroxide (100 μM) and myeloperoxidase (1μg/ml) for 8 or 16 h, and MTT assays were carried out using a Cell Proliferation Kit I (Roche, Indianapolis, IN), according to the manufacturer's instructions. After solubilization of formazan crystals, spectrophotometrical absorbance of samples was measured at 550 nm using a microplate reader. For quantitative RT-PCR, RSL cells were exposed to reagents such as IL-10 (50 ng/ml), CoPP (10 μM), or ZnPP (20 μM) for 6 h, and total RNA was extracted using TRIzol (Life Technologies). After cDNA was synthesized using TaqMan reverse transcription kit (Life Technologies), multiplex PCR was performed using the ABI 7500 Real-Time PCR system (Applied Biosystems, Foster City, CA) with gene-specific primers (FAM-conjugated probes for NRF2, NR0B2, HMOX1, and CCL2) and control primers (a VIC-conjugated probe for GAPDH). The cycle threshold (CT) values were determined according to the manufacturer's instructions. The relative quantity of mRNA was determined using the 2^ΔΔCT^ method (Livak and Schmittgen, 2001). CT values were normalized to the internal control (GAPDH), and the results were expressed as a fold change in mRNA, with the mRNA levels in the non-treated group set as 1. For conventional PCR, primers were used as follows: mouse Bcl-xL (228 bp), 5′-TCCTGGAAGAGAATCGCTAAAC-3′ and 5′-CCCTCTCTGCTTCAGTTTCTT-3′; mouse IL-10R2 (256 bp), 5′-GGACAGGCAATGACGAAATAAC-3′ and 5′-GGGAAGGAGAACAGCAGAAA-3′; mouse IL-22RA (294 bp), 5′-CATGACCTGTTCTACCGCTTAG-3′ and 5′-AGGTGGCTTGGTGATGTATTT-3′; and 18S rRNA (200 bp), 5′-GTGGAGCGATTTGTCTGGTT-3′ and 5′-CGCTGAGCCAGTCAGTGTAG-3′. PCR products were analyzed by electrophoresis on 1.5% agarose gels, viewed after staining with GelRed Nucleic Acid Stain (Biotium, Hayward, CA) and photographed using ChemiDoc (Bio-Rad, Hercules, CA).

### Adenoviral vector, plasmid, transfection, and luciferase assays

To overexpress NR0B2, RSL cells were transfected with the adenoviral vector expressing NR0B2, kindly provided by Dr. Eun-Kyeong Jo (Chungnam National University, South Korea; Yuk et al., 2011). For luciferase assays, RSL cells were transfected with the luciferase-expressing vector with an antioxidant response element (ARE), a gift from Dr. Raekil Park (GIST, South Korea; So et al., 2006), at 60% confluence using the Transit-LT1 transfection reagent (Mirus, Madison, WI), according to the manufacturer′s instructions. The pRL-TK vector (Promega, Madison, WI) was cotransfected to normalize for transfection efficiency. Transfected cells were starved overnight in serum-free DMEM and harvested after exposure to IL-10 (50 ng/ml) for 16 and 42 h. Luciferase activity was measured using a luminometer after adding the necessary luciferase substrate (Promega). Results were expressed as a fold change in luciferase activity, taking the value of the non-treated group as 1.

### Statistics

For technical and independent replicates, all experiments were carried out in triplicate and repeated twice independently. For quantitative RT-PCR analysis and luciferase assays, results were analyzed with the Student *t*-test and one-way ANOVA followed by the Tukey *post-hoc* test using R2.14.0 software for Windows (The R Foundation for Statistical Computing). A *p-*value < 0.05 was considered significant.

## Results

### Hypochlorous acid is involved in cochlear injury

**Figure 1:**
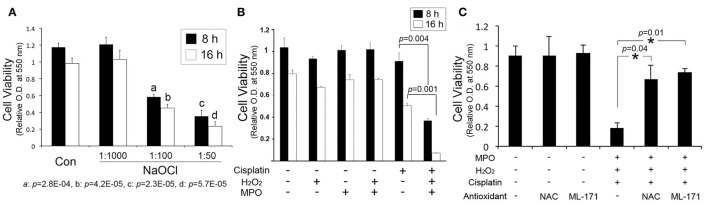
Hypochlorous acid contributes to cochlear injury. **(A)** MTT assays show that sodium hypochlorite (NaOCl) inhibits the viability of HEI-OC1 cells in a dose-dependent manner. **(B)** Combination of hydrogen peroxide (H_2_O_2_) and myeloperoxidase (MPO) augments cisplatin cytotoxicity to HEI-OC1 cells. **(C)** N-Acetyl-L-cysteine (NAC) and ML-171 inhibit the enhancement of cisplatin cytotoxicity by the combination of H_2_O_2_ and MPO. Data are mean ± *SD* (*n* = 3). ^*^p < 0.05.

Hypochlorous acid, generated by myeloperoxidase-mediated peroxidation of chloride ions, is cytotoxic to various mammalian cells such as epithelial cells and red blood cells. To determine whether hypochlorous acid is cytotoxic to cochlear cells *in vitro*, we performed cell viability assays with HEI-OC1, an organ of Corti cell line after exposure to NaOCl, a sodium salt of hypochlorous acid, used as bleaches and deodorants. As shown in Figure 1A, NaOCl reduced cell viability of HEI-OC1 cells in a dose-dependent manner. HEI-OC1 cells were viable in a 1:1000 dilution of NaOCl whereas its 1:100 dilution showed significant cytotoxicity. Furthermore, combination of a recombinant myeloperoxidase with hydrogen peroxide was not cytotoxic to HEI-OC1 cells but appeared to potentiate cisplatin cytotoxicity (Figure 1B). However, hydrogen peroxide or myeloperoxidase alone insignificantly affect cisplatin cytotoxicity (data not shown). Then, we sought to determine an effect of antioxidants on hypochlorous acid-induced cytotoxicity. It was found that hypochlorous acid-mediated augmentation of cisplatin cytotoxicity is inhibited by N-Acetyl-L-cysteine and ML-171 (a NADPH oxidase 1 inhibitor) (Figure 1C). Taken together, it is suggested that hypochlorous acid, when it is excessively released by uncontrolled inflammatory reactions, potentially contributes to induction and exacerbation of cochlear injury.

### NRF2 and NR0B2 contribute to the inhibitory effect of IL-10 on chemokine production in otic fibrocytes

**Figure 2:**
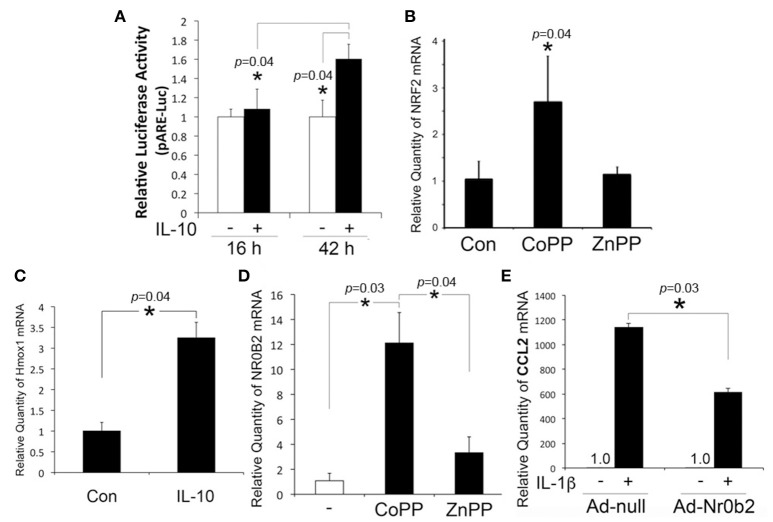
Involvement of NRF2 and NR0B2 in the inhibitory effect of IL-10 on CCL2 regulation. **(A)** Luciferase assays show that IL-10 upregulates ARE activity in RSL cells. Quantitative RT-PCR analysis shows that RSL cells upregulate NRF2 upon exposure to CoPP **(B)** and HMOX1, to IL-10 **(C)**. NR0B2 is upregulated in response to CoPP in RSL cells **(D)**, and transfection of the adenoviral vector expressing NROB2 (Ad-Nr0b2) inhibits IL-1β-induced monocyte chemoattractant protein-1 (CCL2) upregulation **(E)**. Ad-null: a negative control vector. Data are mean ± *SD* (*n* = 3). ^*^p < 0.05.

In our prior study, otic fibrocytes were shown to play a pivotal role in cochlear inflammation (Moon et al., 2007; Oh et al., 2012). It is unclear how IL-10 maintains HMOX1 upregulation in otic fibrocytes despite the inhibition of p38 MAPK required for HMOX1 upregulation (Kontoyiannis et al., 2001). In addition to p38 MAPK, NRF2 appeared to be involved in HMOX1 regulation in HEI-OC1 cells (So et al., 2006), which is a basic leucine zipper protein involved in the regulation of antioxidant genes. Therefore, we investigated an effect of IL-10 on the ARE/NRF2 system in otic fibrocytes. Luciferase assays were conducted with a luciferase vector containing ARE. As shown in Figure 2A, RSL cells appeared to upregulate ARE activity after exposure to IL-10. CoPP (a HMOX1 inducer), not ZnPP (a HMOX1 inhibitor), was found to upregulate NRF2 expression (Figure 2B), and HMOX1 expression was upregulated by IL-10 (Figure 2C). These findings indicate an existence of the NRF2-mediated positive feedback loop in otic fibrocytes, which may be required for maintaining IL-10-induced HMOX1 upregulation, through bypassing the p38 MAPK pathway.

In addition, we sought to determine the involvement of NR0B2 in the inhibitory effect of IL-10 on chemokine production because NR0B2, similar to IL-10 and carbon monoxide (CO) (Woo et al., 2015), suppresses inflammatory reactions through inhibition of NF-κB (Yuk et al., 2011). Quantitative RT-PCR analysis showed that RSL cells upregulate NR0B2 expression ~12-fold in response to CoPP, but ~3-fold to ZnPP (Figure 2D). Furthermore, adenoviral vector-mediated overexpression of NR0B2 was found to inhibit IL-1β-induced CCL2 upregulation in RSL cells (Figure 2E), which suggests the involvement of NR0B2 in the anti-inflammatory effect of IL-10.

### Cochlear localization of IL-10-expressing cells

**Figure 3:**
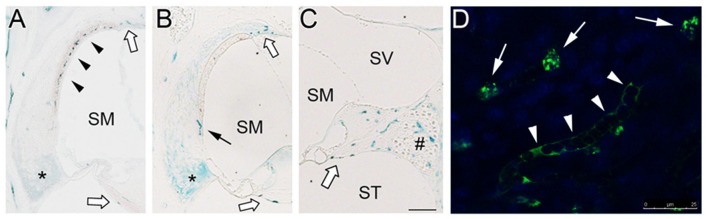
Localization of IL-10-expressing cells in the mouse cochlea. **(A)** Immunolabeling shows appearance of IL-10-positive cells (black arrowheads) in the stria vascularis 1 d after LPS injection. IL-10 labeling is also noted in the lower part of the spiral ligament (asterisk). **(B,C)** On 5 d after LPS injection, IL-10-positive cells are observed in the spiral ligament (black arrow), but not in the stria vascularis. IL-10-positive cells are distributed in the spiral limbus, spiral ganglion area (#), suprastrial area and osseous spiral lamina (white block arrows). SM, scala media; ST, scala tympani; SV, scala vestibule. Scale bar: 100 μm. **(D)** Surface preparation showing IL-10-positive cells (white arrows) in the cochlear lateral wall tissue. IL-10 is also labeled in a capillary-like structure. Scale bar: 25 μm.

Inner ear IL-10 expression is upregulated in experimental tympanogenic cochlear inflammation at the mRNA and protein levels (Trune et al., 2015); however, an intra-cochlear source of IL-10 still remains unclear. To determine cochlear localization of IL-10-expressing cells, immunolabeling of the mouse temporal bone sections were carried out. IL-10-positive cells first appeared in the stria vascularis on 1 d after LPS injection (Figure 3A). Consistently, surface preparation of the cochlear lateral wall showed the localization of IL-10-positive cells in the stria vascularis (Figure 3D). In addition, IL-10 was labeled in the suprastrial area and the lower part of the spiral ligament. On 5 d after injection, IL-10-positive cells were broadly found in the spiral ligament, spiral limbus and spiral ganglion (Figure 3C), but not in the stria vascularis (Figure 3B). IL-10 was not significantly labeled in the saline-injected control mice (data not shown). Altogether, these findings suggest that there are two groups of IL-10-expressing cells in the cochlea: recruited cells and resident cells, but their immunological characteristics remain to be revealed.

### IL-10R expression in the organ of corti cells

**Figure 4:**
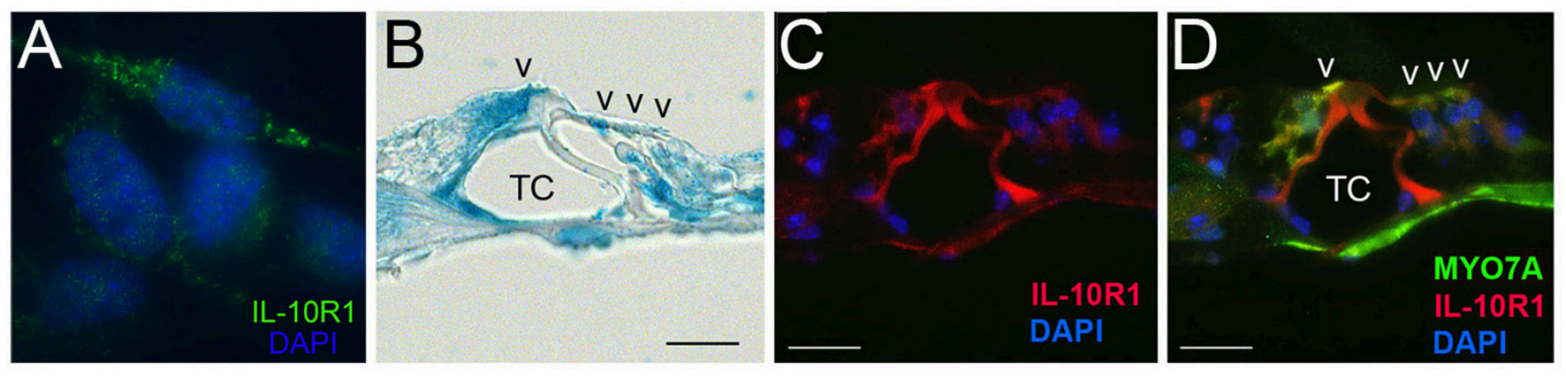
Immunolabeling showing IL-10R1 expression in the HEI-OC1 cells **(A)** and mouse organ of Corti **(B–D)**. Note IL-10R1 labeling in the inner and outer hair cells as well as pillar cells. TC, Tunnel of Corti; V, auditory sensory cells. Scale bar: 25 μm.

The IL-10R complex is a tetramer composed of two subunits, IL-10R1 and IL-10R2. Previously, we have demonstrated the inducible expression of IL-10R1 and constitutive expression of IL-10R2 in otic fibrocytes (Woo et al., 2015), but it is unclear if other cochlear cells express IL-10Rs. To determine whether the organ of Corti cells express IL-10R1 enabling them to respond to IL-10, we performed immunolabeling with an anti-IL-10R1 antibody. As shown in Figure 4A, HEI-OC1 cells appeared to express IL-10R1. Moreover, IL-10R1 labeling was noted in the mouse organ of Corti cells such as inner and outer hair cells as well as pillar cells (Figures 4B–D), but further studies are needed to characterize IL-10R-expressing cells in the organ of Corti.

### IL-10 is protective for cochlear cell injury

**Figure 5:**
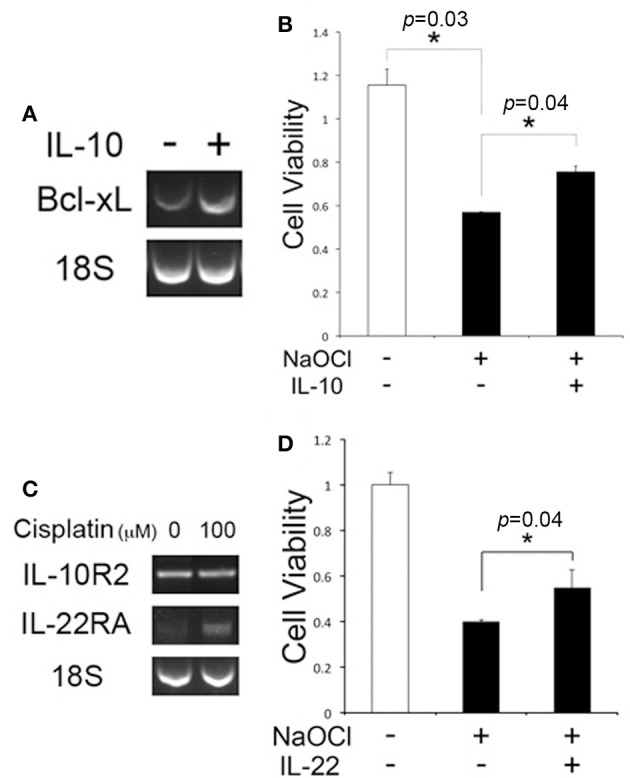
IL-10 and IL-22 are protective against NaOCl cytotoxicity to HEI-OC1 cells. **(A)** Conventional RT-PCR analysis showing the Bcl-xL regulation in HEI-OC1 cells upon exposure to IL-10. 18S: 18S rRNA. **(B)** IL-10 enhances cell viability of HEI-OC1 cells against NaOCl cytotoxicity. **(C)** Conventional RT-PCR analysis showing the IL-22RA expression in cisplatin-treated HEI-OC1 cells. **(D)** MTT assays showing a protective effect of IL-22 on HEI-OC1 cells against NaOCl cytotoxicity. Data are mean ± *SD* (*n* = 3). ^*^p < 0.05.

In addition to the anti-inflammatory function, there is substantial evidence of the cytoprotective effect of IL-10 (Boyd et al., 2003; Zhou et al., 2009), but it is unclear whether IL-10 is protective for cochlear cell injury. HEI-OC1 cells were exposed to IL-10, and RT-PCR analysis was carried out. HEI-OC1 cells appeared to express Bcl-xL higher in response to IL-10 (Figure 5A), but further quantitative analysis would be needed to show IL-10-induced Bcl-xL regulation. MTT assays showed that IL-10 enhances the viability of HEI-OC1 cells against NaOCl-induced cytotoxicity (Figure 5B), but cisplatin cytotoxicity was not affected by IL-10 (data not shown). In addition to IL-10, we sought to determine a cytoprotective activity of IL-22, a member of the IL-10 superfamily because it uses an IL-10R2 subunit for forming an active IL-22R complex. Conventional RT-PCR analysis showed cisplatin-treated HEI-OC1 cells express IL-22RA (Figure 5C). Similar to IL-10, IL-22 was found to reduce NaOCl cytotoxicity to HEI-OC1 cells (Figure 5D). Taken together, it is suggested that IL-10R2 ligands such as IL-10 and IL-22 have a protective effect on cochlear injury, mediated by regulation of anti-apoptotic factors.

## Discussion

Acquired SNHL is associated with postnatal cochlear injuries induced by a variety of causes including ototoxic drugs. This type of hearing loss is clinically important because it is potentially preventable and manageable if cochlear sensorineural tissues are preserved before they are irreversibly damaged. Multiple mechanisms are involved in the damage and survival of the cochlear sensorineural tissue (Wong and Ryan, 2015), and understanding those mechanisms would facilitate the development of a novel clinical tool for the management of acquired SNHL.

Tissue injury, in the absence of infection, is able to trigger inflammatory response to scavenge damaged tissues, but uncontrolled excessive inflammation rather leads to inadvertent tissue injury. Cochlear inflammation is like a double-edged sword; it is either harmful or beneficial to hosts. For instance, cochlear infiltration of macrophages is protective for the survival of spiral ganglion neurons after cochlear sensory cell death (Kaur et al., 2015). On the contrary, cochlear inflammation was found to not only mediate and but also aggravate ototoxic injury (So et al., 2007; Oh et al., 2011; Hirose et al., 2014). In this study, we showed hypochlorous acid, an inflammatory mediator released from activated phagocytes, is able to reduce the viability of cochlear cells *in vitro*. Further animal studies are needed to reveal how inflammatory mediators contribute to inflammation-mediated cochlear injury in a complex *in vivo* system.

Hypochlorous acid is a powerful oxidizing agent reacting with a wide variety of biomolecules such as proteins, nucleotides, and lipids, enabling it to kill pathogens (Pullar et al., 2000). For instance, hypochlorous acid inactivates proteins containing sulfhydryl groups, such as glutathione, by formation of disulfide bonds that result in crosslinking of proteins. Hypochlorous acid also reacts with NADH and the NH-groups of pyrimidines, leading to DNA denaturation. Reacting with lipids, hypochlorous acid induces formation of a chlorohydrin that can disrupt lipid bilayers and increase permeability. Consequently, hypochlorous acid, forming a significant cell stress, reduces the viability of various mammalian cells through apoptotic and necrotic cell death (Yap et al., 2006; Yang et al., 2012). Hypochlorous acid is known to induce Bax-dependent mitochondrial permeabilisation in chondrocytes, resulting in caspase-independent cell death (Whiteman et al., 2007). Similarly, hydrogen peroxide-induced oxidative stress appeared to lead to mitochondrial damage in cochlear sensory cells (Baker and Staecker, 2012), but it is yet to be elucidated how hypochlorous acid induces cochlear sensory cell death. Hypochlorous acid also appeared to enhance cisplatin cytotoxicity to HEI-OC1 cells, and this enhancement was inhibited by antioxidants. These findings suggest the potential involvement of hypochlorous acid-generating leukocytes. In cisplatin nephrotoxicity, there is an extensive renal infiltration of neutrophils (Tadagavadi et al., 2015), but it seems not to be apparent in cisplatin ototoxicity. This may be due to the blood-labyrinth barrier, but further studies are needed to reveal a role of leukocytes in cisplatin ototoxicity.

Otic fibrocytes, representing a heterogeneous population of cells localized in the spiral ligament and limbus, are importantly involved in normal hearing physiology as a route for potassium recycling and the formation of the mechanical anchorage for the basilar and tectorial membranes. In addition, otic fibrocytes were found to play an immunological role in the induction of cochlear inflammation and were able to suppress chemokine production in response to IL-10 (Moon et al., 2007; Oh et al., 2012; Woo et al., 2015). Similarly, circulating fibrocytes, which comprise <0.5% of blood cells, have been implicated in the pathophysiology of various inflammatory diseases through antigen presentation and secretion of inflammatory mediators (Galligan and Fish, 2013). Unlike otic fibrocytes originated from the periotic mesenchyme, circulating fibrocytes are derived from the bone marrow and are frequently transformed to activated fibroblast as observed in rheumatoid arthritis. Bone marrow cells were shown to contribute to the turnover of otic fibrocytes (Lang et al., 2006), but the involvement of circulating fibrocytes remains to be revealed.

**Figure 6:**
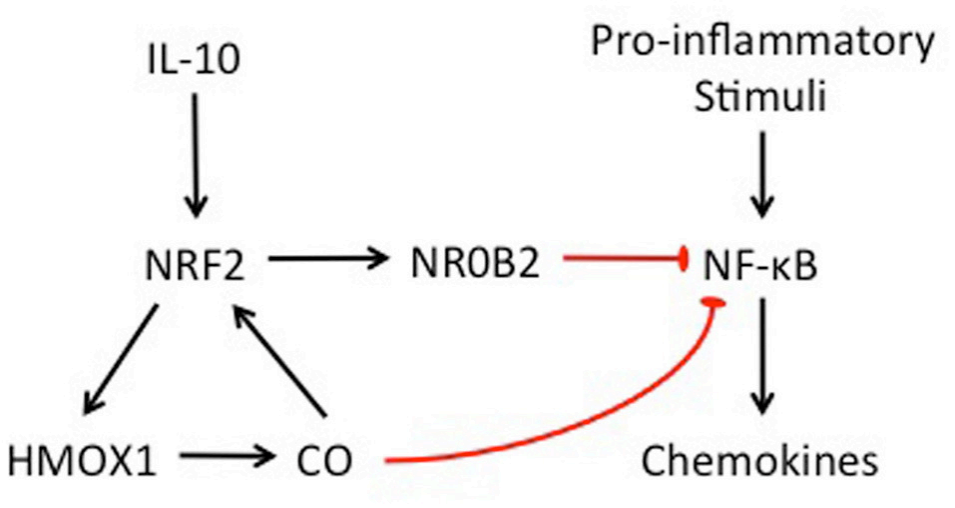
Schematic illustration showing the involvement of NRF2 and NR0B2 in the anti-inflammatory activity of IL-10 in otic fibrocytes.

In this study, we showed that our *in vitro* model of otic fibrocytes preserves the NRF2/CO-mediated feedback loop and CO-mediated NR0B2 regulatory pathway, involved in the anti-inflammatory activity of IL-10 (Figure 6). These findings suggest a therapeutic potential of CO releasers and NR0B2 inducers for the management of inflammation-mediated cochlear injury. For instance, ruthenium-based CO releasers not only suppress inflammatory responses *in vitro* (Sawle et al., 2005) but also ameliorate experimental acute pancreatitis (Xue and Habtezion, 2014). In addition, fenofibrate, a drug clinically used for hyperlipidemia, was found to attenuate experimental sepsis through NR0B2 upregulation (Yang et al., 2013). However, it remains to be revealed whether and how those pharmaceuticals affect inflammation-mediated cochlear injury.

IL-10 is predominantly produced by immune cells such as regulatory T cells, macrophages, and dendritic cells. For decades, the mammalian cochlea has been considered an immune-privileged organ. However, there is evidence supporting the presence of resident and recruited immune cells in the mammalian cochlea (Hirose et al., 2005; Okano et al., 2008). There are at least two types of resident macrophages in the cochlear lateral wall, including cochlear macrophages in the spiral ligament and perivascular macrophage-like melanocytes in the stria vascularis (Okano et al., 2008; Zhang et al., 2012). This study emphasized IL-10 expression in the recruited cells while our previous work showed the IL-10 expression in the isolated mouse cochlear lateral wall tissue, indicating its expression in the resident cells (Woo et al., 2015). Altogether, these findings suggest the involvement of both recruited cells and resident cells in IL-10 production in the cochlea, but further studies are necessary to elucidate their immunological characteristics.

In addition to the anti-inflammatory activity, there is accumulating evidence showing a cytoprotective effect of IL-10 on various mammalian cells such as neuronal cells (Molina-Holgado et al., 2001; Zhou et al., 2009; Lin et al., 2015). Cisplatin-induced acute renal injury also appeared to be reduced by systemic administration of IL-10 (Deng et al., 2001). Consistently, IL-10 deficiency and NRF2 depletion were shown to exacerbate experimental cisplatin nephrotoxicity (Liu et al., 2009; Tadagavadi and Reeves, 2010). In the cochlea, IL-10 has been reported to attenuate autoimmune hearing loss in experimental animals (Zhou et al., 2012). In agreement, we demonstrated the IL-10R1 expression in the organ of Corti as well as IL-10-mediated cytoprotection against hypochlorous acid-induced cytotoxicity. IL-10 also appeared to increase Bcl-xL expression, and Bcl-xL is suggested to inhibit hypochlorous acid cytotoxicity by retrotranslocation of Bax from the mitochondria into the cytosol (Edlich et al., 2011). IL-22, one of the IL-10 family cytokines also showed a meaningful cytoprotective activity, but further studies are needed to determine if combination of IL-10 and IL-22 synergistically upregulates IL-10R2/STAT3-mediated Bcl-xL expression and is effective for more potent ototoxic drugs. Moreover, animal studies are required to reveal how IL-10 affects cochlear inflammation and injury *in vivo*. Altogether, our results indicate the feasibility of an IL-10-based approach to manage inflammation-mediated cochlear injury.

Collectively, it is suggested that the IL-10/IL-10R axis is crucially involved in the modulation of cochlear inflammation in otic fibrocytes and the protection against inflammation-mediated cochlear injury in the organ of Corti. Understanding the complex network in IL-10 signaling would provide a new therapeutic target for the management of cochlear injury resulting in acquired SNHL.

## Author contributions

MM and SK carried out the majority of the experiments and equally contributed to this work. DP and HP were involved in cell viability assays, immunolabeling, and statistical analysis. DL and RP were involved in histological analysis, data analysis, and preparation of the manuscript. SM designed the experiments, analyzed the results, and prepared the manuscript.

### Conflict of interest statement

The authors declare that the research was conducted in the absence of any commercial or financial relationships that could be construed as a potential conflict of interest.
